# The Effects of Exogenous Lactate Administration on the IGF1/Akt/mTOR Pathway in Rat Skeletal Muscle

**DOI:** 10.3390/ijerph17217805

**Published:** 2020-10-25

**Authors:** Sunghwan Kyun, Choongsung Yoo, Hun-Young Park, Jisu Kim, Kiwon Lim

**Affiliations:** 1 Department of Physical Education, Konkuk University, Gwangjin-gu, Seoul 05029, Korea; y10345kyun@naver.com; 2 Department of Health and Kinesiology, Texas A&M University, College Station, TX 77843, USA; choongsungyoo@tamu.edu; 3 Department of Sports Medicine and Science, Konkuk University, Gwangjin-gu, Seoul 05029, Korea; parkhy1980@konkuk.ac.kr (H.-Y.P.); kimpro@konkuk.ac.kr (J.K.); 4 Physical Activity and Performance Institute (PAPI), Konkuk University, Gwangjin-gu, Seoul 05029, Korea

**Keywords:** exogenous lactate, protein synthesis, protein degradation, Akt, mTOR, IGF receptor

## Abstract

We investigated the effects of oral lactate administration on protein synthesis and degradation factors in rats over 2 h after intake. Seven-week-old male Sprague–Dawley rats were randomly divided into four groups (*n* = 8/group); their blood plasma levels of lactate, glucose, insulin, and insulin-like growth factor 1 (IGF1) were examined following sacrifice at 0, 30, 60, or 120 min after sodium lactate (2 g/kg) administration. We measured the mRNA expression levels of protein synthesis-related genes (IGF receptor, protein kinase B (Akt), mammalian target of rapamycin (mTOR)) or degradation-related genes (muscle RING-finger protein-1 (MuRF1), atrogin-1) and analyzed the protein expression and phosphorylation (activation) of Akt and mTOR. Post-administration, the plasma lactate concentration increased to 3.2 mmol/L after 60 min. Plasma glucose remained unchanged throughout, while insulin and IGF1 levels decreased after 30 min. The mRNA levels of IGF receptor and mTOR peaked after 60 min, and Akt expression was significantly upregulated from 30 to 120 min. However, MuRF1 and atrogin-1 expression levels were unaffected. Akt protein phosphorylation did not change significantly, whereas mTOR phosphorylation significantly increased after 30 min. Thus, lactate administration increased the mRNA and protein expression of protein-synthesis factors, suggesting that it can potentially promote skeletal muscle synthesis.

## 1. Introduction

Approximately 40–50% of human body weight consists of skeletal muscle, which is characterized by plasticity. Skeletal muscle mass is determined by the balance between synthesis and degradation rates [1,2]. Thus, stimulating synthesis factors have an important effect on increasing muscle mass. Muscle synthesis is induced by a number of factors in the body, among which the insulin-like growth factor 1 (IGF1)/protein kinase B (Akt)/mammalian target of rapamycin (mTOR) pathway has directly been shown to increase protein synthesis [3,4,5]. The IGF1/Akt/mTOR pathway regulates cell proliferation and differentiation in adult skeletal muscle [6]. In addition, IGF1/Akt/mTOR pathway activity can also inactivate protein degradation factors such as muscle RING-finger protein-1 (MuRF1) and muscle-specific F-box protein (atrogin-1) [6,7,8]. Indeed, Akt and mTOR phosphorylation upregulated in C2C12 cell myotubes to which IGF1 was administered, and the myotube diameter increased compared to that of the control group [3]. When rapamycin, a selective mTOR inhibitor, was injected into rats with compensatory hypertrophy, their muscle mass was lower than that in similar untreated rats [6]. Moreover, in C2C12 myotubes with induced atrophy, IGF1 treatment (10 ng/mL) decreased the expression of the atrophy markers atrogin-1 and MuRF1, increasing the myotube diameter [7]. Therefore, to increase the muscle mass, it is necessary to regulate the balance between the factors driving protein synthesis and degradation.

Lactate has been traditionally recognized as a fatigue-associated molecule because early reports indicated that lactate is produced by anaerobic glycolysis and induced muscular acidosis [9,10]. However, the understanding of lactate has changed in recent decades, and its roles as an efficient energy resource [11,12] and a potential factor associated with exercise-induced muscle adaption [13] have been reconsidered.

Interestingly, several recent studies have shown positive effects of lactate on protein synthesis. In vitro, Ohno et al. [14] reported that C2C12 cells treated with 20 mM lactate had increased protein content and myonucleus number. Oishi et al. [15] also reported the effects of lactate-based supplementation on C2C12 cells. In their study, treatment with 10 mM lactate and 5 mM caffeine for 6 h increased the satellite cell activity and anabolic signals. In vivo, acute intraperitoneal injection of lactate (3 g/kg) increased the blood lactate levels to approximately 20 mmol/L after 5 min, and increased the phosphorylation of Akt (Thr308 and Ser473) and ribosomal protein S6 kinase beta-1 (p70S6K), which is downstream of the IGF1/Akt/mTOR-pathway, in the quadriceps muscle after 40 min [16]. In addition, 1 g/kg lactate administrated mice showed a supplemental effect on tibialis anterior weight and muscle fiber cross-sectional area [14]. These findings revealed that lactate administration might increase the levels of factors driving protein synthesis.

However, to date, little research has been conducted regarding the effects of lactate on muscles. Before using lactate as a dietary supplement, the effects of its oral administration on muscles need to be confirmed. Most studies have involved in vitro treatment, although some in vivo studies have employed intraperitoneal injection for lactate supplementation. Moreover, most previous studies have focused only on the effect of lactate on protein synthesis factors. However, a recent study reported that lactate overload may cause muscular atrophy in C2C12 myotubes [17]. Finally, data from previous studies have shown that exogenous lactate can increase blood lactate concentrations over time [14,16,18]. Nevertheless, no studies of the changes in protein synthesis factors following lactate administration over time (e.g., 0, 30, 60, and 120 min) have been reported. Because the timing of lactate administration is important for its use as a supplement, it is necessary to confirm the relevant changes over time.

The central hypothesis of this study was that exogenous lactate administration produces different responses in a time-dependent manner and can induce protein synthesis. To test this hypothesis, we administered lactate to rats, analyzed the mRNA and protein expression of protein synthesis- and protein degradation-related factors in skeletal muscles, and evaluated the blood factors in plasma.

## 2. Materials and Methods

### 2.1. Animal Care

The animals were cared for as previously reported [19,20,21]. Male Sprague–Dawley rats (7 weeks of age) were obtained from Raon Bio (Korea). The rats were housed in standard cages under controlled conditions (50% humidity, 23 ± 1 °C, and an alternating 12 h light–dark cycle). The rats were fed ad libitum with a commercial normal diet (65% carbohydrate, 12% fat, and 14% protein). The animal experiment was conducted as per the ethical guidelines of the Animal Experiment Research Center of Konkuk University. This study was also conducted in accordance with the ethical guidelines of the Konkuk University Institutional Animal Care and Use Committee (No. KU18137).

### 2.2. Animal Protocol

**Figure 1:**
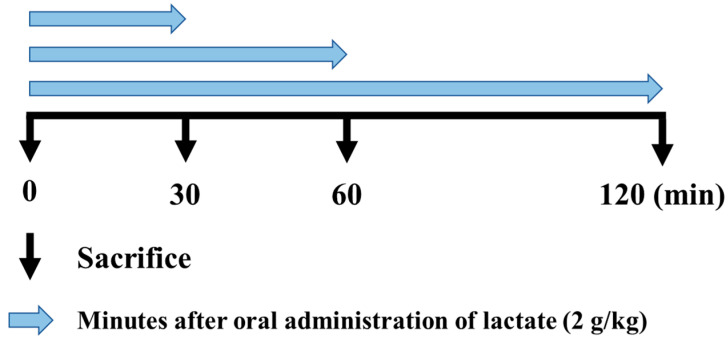
Study design. Blood and muscle samples were obtained at the indicated time points after oral lactate administration to analyze mRNA expression levels and blood (plasma) parameters.

Thirty-two rats were used in the study. They were randomly divided into 4 groups that were sacrificed at 0 min (control group), 30 min, 60 min, or 120 min after lactate administration with an oral sonde (Figure 1) at a concentration of 2 g/kg [22,23]. All rats were fasted for 2 h before lactate administration and were euthanized by vertebral dislocation at the predetermined time points. Immediately after sacrifice, 3 mL blood samples were obtained from the arteries, and skeletal muscle tissues (plantaris) were quickly removed, frozen in liquid nitrogen, and stored at −80 °C until analysis.

### 2.3. Blood Parameters

The arterial blood samples were immediately collected into ethylenediaminetetraacetic acid (EDTA) tubes. After 30 min, the blood samples were centrifuged at 2000× *g* for 15 min at 4 °C, and the collected plasma was stored at −80 °C. We analyzed the blood (plasma) concentrations of lactate (K607; Bio Vision, USA), insulin (80-INSRT-E01; ALPCO, Salem, NH, USA), IGF1 (ab231924; Abcam, Cambridge, UK), and glucose (K039-H1; Arbor Assays, Ann Arbor, MI, USA.), using the indicated colorimetric kits.

### 2.4. Reverse Transcription-Polymerase Chain Reaction (RT-PCR) Analysis

We performed RT-PCR to determine the expression levels of mRNAs of interest. The mRNA obtained from the right plantaris was used to measure the expression levels of glyceraldehyde 3-phosphate dehydrogenase (GAPDH) and five other factors. Total RNA was extracted from the plantaris using QIAzol Lysis Reagent (79306; Qiagen, Hilden, Germany). We synthesized complementary DNA (cDNA) from total RNA using amfiRivert cDNA Synthesis Platinum Master Mix (R5600; GenDEPOT, Katy, TX, USA). We then performed the RT reaction using the following protocol: annealing for 5 min at 25 °C, extension for 50 min at 42 °C, and RT inactivation for 15 min at 70 °C. For RT-PCR, cDNA was amplified with amfiEco Taq DNA polymerase (P0701; GenDEPOT, Katy, TX, USA) and the following primer pairs: IGF receptor-F and -R; Akt-F and -R; mTOR-F and -R; MuRF1-F and -R; and atrogin-1-F and -R (Table 1). We then examined the expression levels of the protein synthesis-related factors IGF receptor, Akt, and mTOR, and the protein degradation-related factors MuRF1 and atrogin-1. The cycling conditions were as follows: an initial denaturation for 2 min at 94 °C, followed by 25–40 cycles of 15 s at 94 °C, 30 s at 50–70 °C, and 1 min at 72 °C. Finally, we separated the products on 1% agarose gels and visualized them using the Safe-Pinky DNA gel-staining solution (S1001-025; GenDEPOT, Katy, TX, USA).

### 2.5. Western Blotting

Western blotting was performed to investigate the protein levels in the plantaris muscle. Muscles obtained from rats in the respective groups (0, 30, 60, 120 min) were homogenized in 250 μL of protein extraction buffer (EzRIPA Lysis kit, ATTO, Tokyo Japan). After 30 min of incubation on ice, the lysates were centrifuged at 14,000× *g* at 4 °C for 15 min. Protein quantification was performed using a BCA assay kit (Thermofisher, Waltham, MA, USA). Equal amounts of the proteins (20 μg) were separated on 10% SDS–PAGE gels and transferred on to polyvinylidene difluoride membranes. After 1 h of blocking with 5% skim milk, the membranes were incubated overnight (~16 h) at 4 °C with primary antibodies against Akt (1:1000, #4691, Cell Signaling Technology, Danvers, MA, USA), mTOR (1:1000, #2983, Cell Signaling Technology, Danvers, MA, USA), p-Akt (1:1000, #4060, Cell Signaling Technology, Danvers, MA, USA), p-mTOR (1:1000, #5536, Cell Signaling Technology, Danvers, MA, USA), and GAPDH (1:1000, sc-35062, Santa Cruz Biotechnology, Dallas, TX, USA) with 3% skim milk. Then, the membranes were incubated with the secondary antibodies (1:2000, anti-mouse, sc-516102; 1:2000, anti-rabbit, sc-2357; Santa Cruz Biotechnology, Dallas, TX, USA) in 3% skim milk for 2 h at room temperature. Immunoblots were visualized using an enhanced chemiluminescence (ECL) reagent (Amersham Bioscience, Piscataway, NJ, US). All figures showing the results of the quantitative analysis were assessed using Image J software, and the intensity of the analyzed bands were normalized using GAPDH as the reference.

### 2.6. Statistical Analysis

All data were analyzed using the IBM SPSS statistics software package (version 25). Statistical differences in the IGF receptor, Akt, mTOR, MuRF1, and atrogin-1 mRNA-expression levels, as well as the protein levels of Akt and mTOR phosphorylation and the levels of different blood (plasma) components (lactate, insulin, IGF1, and glucose) were determined by one-way analysis of variance, followed by Bonferroni’s test. Probability values of <0.05 were considered to reflect statistically significant differences.

## 3. Results

### 3.1. Blood Concentrations of Lactate, Glucose, Insulin, and IGF1

**Figure 2:**
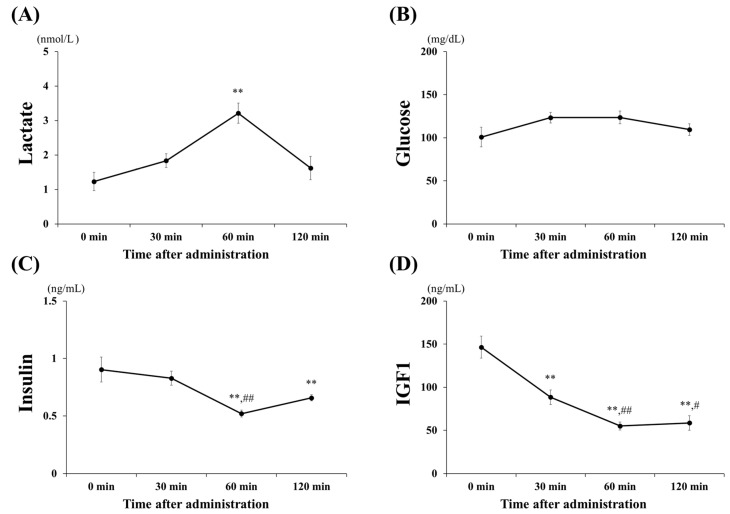
Blood (plasma) parameters. Changes in blood (plasma) (**A**) lactate, (**B**) glucose, (**C**) insulin-like growth factor 1 (IGF1), and (**D**) insulin concentrations after lactate administration (2 g/kg) within 120 min. The values shown represent the means ± SDs (*n* = 8). ** *p* < 0.01, vs. the 0 min (control group). # *p* < 0.05, ## *p* < 0.01, vs. the 30 min after lactate administration.

The blood (plasma) concentrations of lactate, glucose, insulin, and IGF1 following oral administration of lactate are shown in Figure 2. Lactate levels (Figure 2A) significantly increased, to approximately 3.2 mmol/L at 60 min (60 min vs. 0, 30, 120 min; all *p* < 0.001) and returned to baseline at 120 min. Glucose and insulin levels (Figure 2B,C) were investigated to assess whether the administration of lactate affected the blood. Glucose levels did not change significantly within 120 min after lactate administration. However, insulin levels significantly decreased at 60 min (*p* < 0.001) and 120 min (*p* < 0.01) when compared with those at 0 min. The IGF1 levels (Figure 2D), related to the stimulation of anabolic effects, were significantly downregulated, beginning at 30 min post-lactate administration (60 min vs. 0, 30, 120 min; *p* < 0.001).

### 3.2. mRNA Expression of Protein Synthesis and Degradation Factors

**Figure 3:**
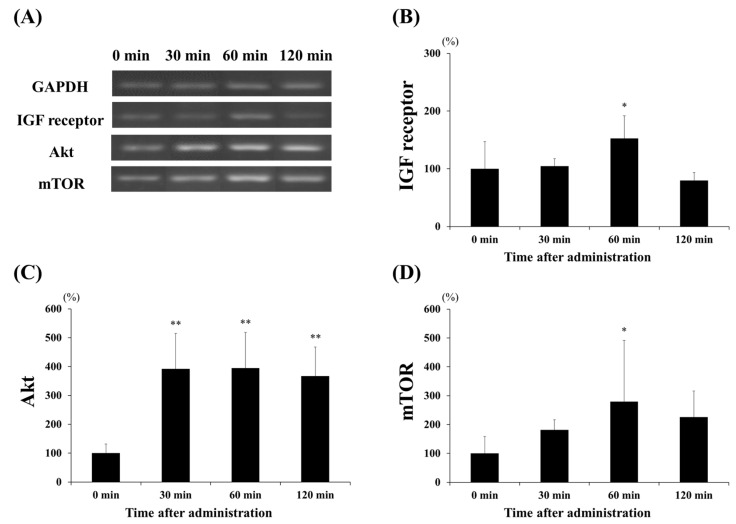
Effect of lactate administration on the expression levels of genes related to protein synthesis. (**A**) Reverse transcription-polymerase chain reaction (RT-PCR) showing the mRNA expression levels of genes related to protein synthesis. (**B**) IGF receptor expression. (**C**) Protein kinase B (Akt) expression. (**D**) Mammalian target of rapamycin (mTOR) expression. Glyceraldehyde 3-phosphate dehydrogenase (GAPDH) was used as a reference and was used to normalize the expression levels of the target genes. The experimental groups included the control group (0 min) and three groups studied 30, 60, or 120 min after lactate administration (2 g/kg). The values shown represent the means ± SDs (*n* = 8). * *p* < 0.05, ** *p* < 0.01, vs. the 0 min (control group).

**Figure 4:**
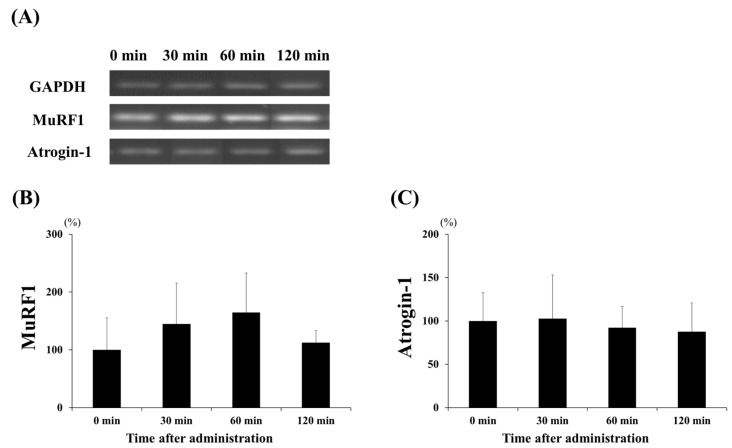
Effect of lactate administration on the expression levels of genes related to protein degradation. (**A**) Reverse transcription-polymerase chain reaction (RT-PCR) bands related to protein degradation. (**B**) Muscle RING-finger protein-1 (MuRF1) expression. (**C**) Muscle-specific F-box protein (atrogin-1) expression. Glyceraldehyde 3-phosphate dehydrogenase (GAPDH) was used as a reference mRNA and was used to normalize the expression of the target genes. The experimental groups included the control group (0 min) and three groups studied 30, 60, or 120 min after lactate administration (2 g/kg). The values shown represent the means ± SDs (*n* = 8). The letters indicate significant differences between the groups (*p* < 0.05).

To investigate the effect of lactate administration on skeletal muscles, we analyzed the mRNA levels of the protein synthesis markers, namely the IGF receptor, Akt, and mTOR (Figure 3); these showed increased expression levels. Figure 3B shows that lactate administration significantly increased IGF receptor expression at 60 min compared to that at the other times (60 min vs. 0 min and 30 min, *p* < 0.05; 60 min vs. 120 min, *p* < 0.001). Akt expression (Figure 3C) significantly increased at 30, 60, and 120 min (*p* < 0.001, in each case), three- to four-fold, when compared to that at 0 min. Moreover, the expression of mTOR (Figure 3D) was significantly higher at 60 min than at 0 min (*p* < 0.05). We also examined the expression levels of two major factors, MuRF1 and atrogin-1, to confirm the effects of lactate administration on protein degradation (Figure 4). However, no significant changes in the expression of the factors related to protein degradation were observed at any time point.

### 3.3. Protein Expression of Akt and mTOR

**Figure 5:**
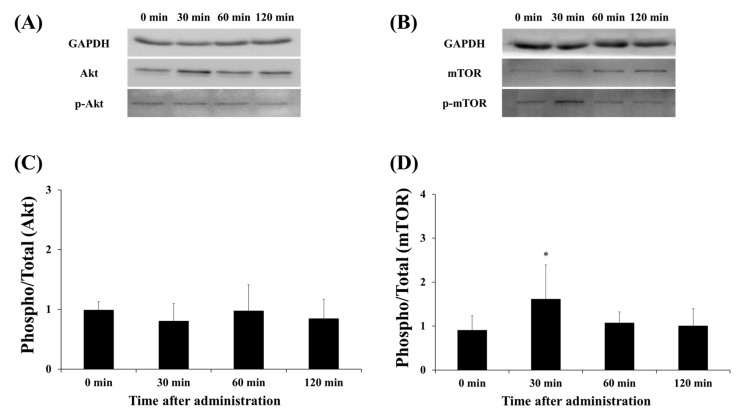
Effect of lactate administration on the protein expression of Akt and mTOR. (**A**,**B**) Western blot bands of Akt and mTOR. (**C**) The ratio of phosphorylated Akt to total Akt at each time point. (**D**) The ratio of phosphorylated mTOR to total mTOR at each time point. The experimental groups included the control group (0 min) and three groups studied 30, 60, or 120 min after lactate administration (2 g/kg). The values shown represent the means ± SDs (*n* = 8). * *p* < 0.05, vs. the 0 min (control group).

Akt and mTOR are factors that are activated via phosphorylation. The effect of the lactate administration on Akt and mTOR total expression and phosphorylation was detected by Western blot analysis (Figure 5). Lactate administration did not change Akt phosphorylation at any time point (Figure 5C). Interestingly, mTOR phosphorylation significantly increased after 30 min, compared with that at the 0-min time point (30 min vs. 0 min, *p* < 0.05).

## 4. Discussion

In this study, we investigated changes in the expression levels of factors related to protein synthesis and degradation at different time points (0, 30, 60, and 120 min) after lactate administration. The main findings were that exogenous lactate increased the mRNA expression of protein synthesis factors (IGF receptor, Akt, and mTOR) and phosphorylation of mTOR, but did not alter the mRNA expression of protein degradation factors (MuRF1 and atrogin-1). In addition, blood (plasma) parameters (namely the lactate, insulin, and IGF1 concentrations) were affected by lactate administration.

Lactate production gradually increases in a manner depending on the intensity of exercise [10]. However, we artificially increased the level of lactate in the plasma using oral lactate administration and compared the hormonal responses related to IGF1 and insulin. Previous data have shown that lactate injection increased plasma lactate levels by approximately 20 mmol/L after 15 min and enhanced protein synthesis factors, such as Akt or myogenin [16,18]. Moreover, in another treatment study, the oral intake of lactate (1 g/kg) also upregulated the plasma lactate concentration to approximately 4.1 mmol/L after 2 h, and increased tibialis anterior weight [14]. We also found that the plasma lactate levels increased to approximately 3.2 mmol/L at 60 min after oral administration of lactate and that the expression of protein synthesis factors increased. Our results were thus similar to published results in terms of increased protein synthesis factors, although the plasma lactate concentration increased more slowly and to a lesser extent after oral lactate administration than after lactate injection.

In addition, our results showed that IGF1 and insulin levels decreased after lactate administration. Insulin is directly involved in glucose storage in muscles, and IGF1 (which is closely related to insulin [24]) has been associated with increased cell proliferation and differentiation [25] and protein synthesis in satellite cells [26]. Morville et al. [27] reported that the plasma insulin concentration was 41.1 ± 4.2 pg/mL before exercise and that it decreased to 19.9 ± 7.2 pmol/L after the 70% VO_2_ peak after 60 min of cycling. Moreover, Boisseau et al. [28] reported that moderate-intensity exercise (30 min of cycling exercise at 60% VO_2_ max) decreased the plasma insulin levels in young men. Furthermore, previous data showed that plasma and skeletal muscle IGF1 levels decreased significantly and that Akt expression increased in muscles after acute aerobic exercise (running on a treadmill at 25 m/min for 60 min) [29]. Based on these results, we hypothesized that oral lactate administration induced a plasma response of IGF1 and insulin similar to that following low- or moderate-intensity exercise and confirmed that the blood reaction began to appear mainly after 30 to 60 min.

In this study, we confirmed that lactate administration increased the mRNA expression of IGF receptor, Akt and mTOR in skeletal muscle. Moreover, we found that mTOR protein phosphorylation was increased. IGF receptors allow the influx of the IGF1 hormone into muscles [25]. Hugo et al. [16] reported that lactate injection did not alter IGF receptor phosphorylation in three different rat skeletal muscle types after 40 min. However, we investigated the results over a longer period after lactate administration and found that IGF receptor mRNA expression increased at 60 min after lactate intake. Thus, a greater influx of the IGF hormone is inducible in this time frame. In addition, we propose that the IGF1 levels in the plasma decreased because of increased intramuscular influx.

Additionally, previous studies have confirmed that lactate intake upregulates protein synthesis factors in the Akt/mTOR-pathway [15,16] and tends to increase muscle mass [14]. However, the difference in our study was that the effect of lactate administration was confirmed over time (within 2 h) and showed that not only was expression of Akt and mTOR mRNA upregulated by 30 to 120 min, but that mTOR phosphorylation also increased after 30 min. These results indicate that lactate administration can induce protein synthesis factors and provide insights into the effects of using lactate intake as a supplement over time.

Furthermore, studies on the effects of lactate on protein degradation have been insufficient to date. Increases in the expression of MuRF1 and Atrogin-1 are associated with atrophy or protein degradation, and have a significant effect in sarcopenia [30]. A recent study on lactate by Oh et al. [17] revealed that 8mM lactate-overloaded C2C12 myotubes had decreased Akt phosphorylation and increased atrogin-1 expression and myotube diameters. However, the results of this study showed that lactate administration did not affect the expression of factors related to protein degradation within 120 min after lactate intake. We suggest that these differences may reflect analysis at different times after lactate administration. Accordingly, we suggest that oral lactate administration increases protein synthesis factors without affecting protein degradation factors.

Although positive effects of lactate administration were observed in skeletal muscles, the present study had some limitations. Previous studies of the effects of lactate mainly used a lactate dosage of 1 to 3 g/kg [14,15,22,31], and we used a lactate dosage of 2 g/kg in this study. Some studies involved the use of lactate-based supplements or combined treatment with exercise [15,31], whereas we used a higher lactate concentration to investigate the effectiveness of lactate treatment alone and confirmed significant positive effects with regard to protein synthesis. However, further studies with various dosages are thus necessary, considering that the effect of lactate dosage remains unclear. Furthermore, based on our study, it is difficult to determine the mechanism(s) driving protein synthesis, because we only used a few methods and confirmed a few factors. In addition, although we observed increased expression of protein synthesis factors shortly after lactate administration, we did not determine whether these results correlated with stable increased skeletal muscle mass after lactate treatment. Further studies are therefore needed to investigate the long-term effects of lactate administration and to confirm the mechanisms, using a variety of methods and investigating additional factors related to protein synthesis.

## 5. Conclusions

**Figure 6:**
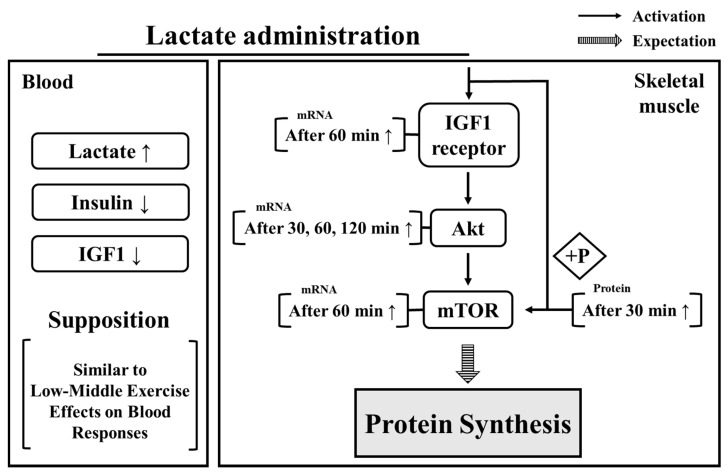
Effect of lactate administration on blood parameters and skeletal muscle. IGF: insulin growth factor 1. IGF receptor: insulin growth factor receptor. Akt: protein kinase B. mTOR: mammalian target of rapamycin.

In summary (Figure 6), the present findings demonstrate that lactate administration had positive effects, i.e., upregulated the expression levels of, protein synthesis factors after 30 min and that the levels of these components remained high for 2 h. Moreover, the reduction of insulin and IGF1 levels, and the upregulation of blood lactate confirmed that the effects of oral lactate administration appear similar to the blood responses observed in humans during exercise. Therefore, our results suggest that consuming lactate as a supplement might induce an increase in the expression of protein synthesis factors. In addition, the period between 30 and 60 min after lactate administration showed the most significant effect on protein synthesis factors and can be used as a reference for timing the effects of lactate intake. Despite these results, because our study focused on the acute effects after lactate intake, further research is required to investigate the long-term effects of lactate administration on protein synthesis and muscle mass.

## Figures and Tables

**Table 1:** Sequences of primers used for reverse transcription-polymerase chain reaction analysis. The primer sequences used to amplify the insulin-like growth factor 1 (IGF-1) receptor, protein kinase B (Akt), the mammalian target of rapamycin (mTOR), muscle RING-finger protein-1 (MuRF1), and muscle-specific F-box protein (atrogin-1) mRNA in muscles were confirmed by RT-PCR.

Gene	Sequences
IGF receptor	F–5′ GAG AAC AAT GAG TGC TGC CA 3′
	R–5′ ACC CTT GGA GCA TCT GGG CA 3′
Akt	F–5′ TGC TGG AGG ACA ACG ACT AT 3′
	R–5′ TGT CAT CTT GAT CAG GCG GT 3′
mTOR	F–5′ TTG AGG TTG CTA TGA CCA GAG AGA A 3′
	R–5′ TTA CCA GAA AGG ACA CCA GCC AAT G 3′
MuRF1	F–5′ ACA TCT TCC AGG CTG CCA AT 3′
	R–5′ GTT CTC CAC CAG CAG GTT CC 3′
Atrogin-1	F–5′ GAC TGG ACT TCT CGA CTG CC 3′
	R–5′ GAC TTG CCG ACT CTC TGG AC 3′
